# Rare case of cartilaginous choristoma of the tongue: case report

**DOI:** 10.21142/2523-2754-1403-2026-308

**Published:** 2026-07-10

**Authors:** Misael Parra Montiel, Ailyn Quirós Jiménez, Yadira V Boza Oreamuno

**Affiliations:** 1 Faculty of Dentistry, University of Costa Rica. San Jose, Costa Rica. misapm2@gmail.com ailynqui005@gmail.com yadira.boza@ucr.ac.cr Universidad de Costa Rica Faculty of Dentistry University of Costa Rica San Jose Costa Rica misapm2@gmail.com ailynqui005@gmail.com yadira.boza@ucr.ac.cr

**Keywords:** choristoma, cartilage, tongue, oral neoplasms, biopsy, coristoma, cartílago, lengua, neoplasias orales, biopsia

## Abstract

**Introduction:**

Choristomas are proliferations of histologically normal tissue occurring in atypical anatomical locations. Cartilaginous choristomas within the oral cavity are rare, most commonly found on the tongue. Clinically, they present as firm, slow-growing, and generally asymptomatic nodules, which may be mistaken for lipomas, fibromas, or other benign soft tissue lesions.

**Case Report:**

We report the case of a 74-year-old woman presenting with a 1 × 1 cm nodule located at the junction of the dorsum and left lateral border of the tongue, with a four-year history. The lesion was managed through complete surgical excision. Histopathological examination confirmed the diagnosis of a cartilaginous choristoma. Over a five-year follow-up period, based on intraoral clinical examinations included visual inspection, palpation, and assessment of tongue mobility, strength, and sensation. No recurrence was observed, the lingual musculature remained unaffected, and the patient retained full motor and sensory function of the tongue.

**Conclusions:**

Lingual cartilaginous choristomas are rare benign lesions that should be considered in the differential diagnosis of submucosal tongue nodules. Familiarity with the clinical presentation and histopathological features of this entity is essential for accurate diagnosis and management by dental professionals.

## RESUMEN

**Introducción:**

Los coristomas son proliferaciones de tejido histológicamente normal en localizaciones anatómicas atípicas. Los coristomas cartilaginosos en la cavidad oral son raros y aparecen con mayor frecuencia en la lengua. Clínicamente, se presentan como nódulos firmes, de crecimiento lento y generalmente asintomáticos, que pueden ser confundidos con lipomas, fibromas u otras lesiones benignas de tejidos blandos.

**Reporte de caso:**

Se presenta el caso de una mujer de 74 años que desarrolló un nódulo de 1 × 1 cm de diámetro en el límite del dorso y el borde lateral izquierdo de lengua, con evolución de cuatro años. La lesión fue tratada mediante remoción quirúrgica completa. El estudio histopatológico confirmó el diagnóstico de coristoma cartilaginoso. Durante un seguimiento de cinco años, basado en exámenes clínicos intraorales que incluyeron inspección visual, palpación y evaluación de la movilidad, fuerza y sensibilidad de la lengua, no se observó recurrencia ni afectación de la musculatura lingual; la paciente conservó la función motora y sensitiva completa de la lengua**.**

**Conclusiones:**

Los coristomas cartilaginosos linguales son lesiones benignas infrecuentes que deben considerarse en el diagnóstico diferencial de nódulos linguales submucosos. Es de suma importancia que los odontólogos se familiaricen con los aspectos clínicos de esta lesión.

## INTRODUCTION

A choristoma is defined as the presence of histologically normal tissue located in an anatomical site where it is not ordinarily found ^1^^, (^^2^. When this ectopic tissue forms a well-circumscribed mass composed of mature cartilage, the lesion is termed a cartilaginous choristoma ^1^.

In contrast, a chondroma is an uncommon benign neoplasm consisting of mature hyaline cartilage that typically arises in regions of cartilaginous ossification, such as the extremities or craniofacial skeleton ^3^^,^^4^.

In the oral cavity, although soft-tissue chondromas have been reported, they are typically found in regions adjacent to bone, such as the gingiva or palate, where cartilaginous remnants may persist or chondrogenic potential may exist ^3^^,^^5^. In contrast, cartilaginous lesions occurring in sites distant from bony structures, such as the tongue or buccal mucosa, are more accurately classified as cartilaginous choristomas, as they consist of histologically normal ectopic cartilaginous tissue rather than a true chondral neoplasm ^1^^,^^6^.

Although cartilaginous choristomas of the oral cavity are rare, they have been reported to occur more frequently on the tongue than in other regions of the oral mucosa ^7^. These lesions typically present as well-circumscribed, firm, slow-growing, and generally asymptomatic nodules-features that can lead to diagnostic confusion with more common entities ^1^^,^^6^. On the tongue, the primary clinical differential diagnoses include traumatic fibroma, lipoma, leiomyoma, neurofibroma, and granular cell tumor ^8^^-^^10^.

Histopathological examination is essential for establishing a definitive diagnosis and excluding lesions of mesenchymal or neoplastic origin ^6^^,^^7^^,^^11^. The treatment of choice is complete surgical excision. According to the literature, cartilaginous choristomas of the tongue and other oral soft tissues are benign lesions that generally show an excellent long term prognosis after complete excision, with no recurrences reported in most case series and reviews ^7^. These findings support complete surgical excision and clinical follow-up.

The objective of this paper is to present a rare case of cartilaginous choristoma of the tongue, detailing its clinical manifestations, histopathological characteristics, surgical management, and follow-up.

## CASE REPORT

A 74-year-old Costa Rican woman from Heredia presented with the chief complaint, “I have a lump on my tongue.” She reported a four-year history of the lesion, which had gradually increased to its current size. Her medical history was significant for an appendectomy, penicillin allergy, atopic dermatitis, and osteopenia, for which she had been treated with Bonviva (ibandronic acid) 150 mg once monthly for five years. At the time of presentation, she was not taking any medications and denied alcohol consumption or smoking.

**Figure 1:**
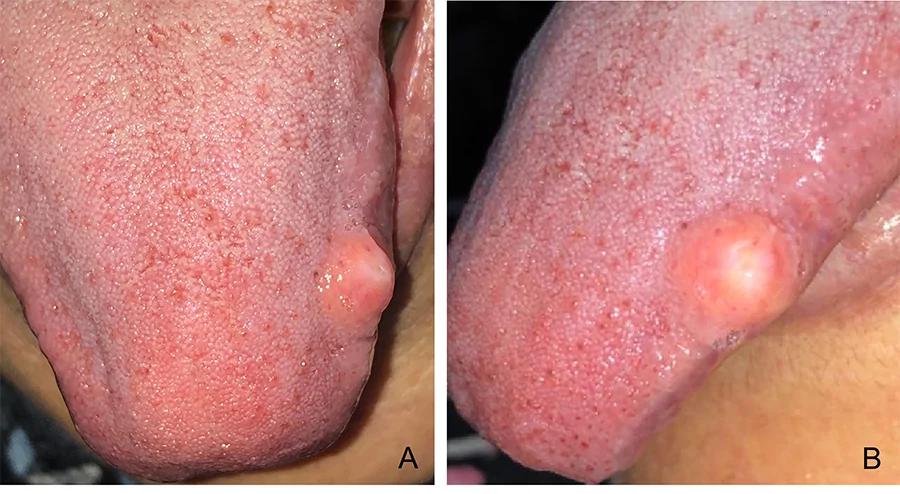
Nodular lesion on the left lateral border of the tongue measuring approximately 1 cm in diameter. (A) Frontal view. (B) Lateral view.

During the intraoral clinical examination, a symmetrical nodular lesion was observed at the junction of the dorsum and the left lateral border of the tongue, extending across the anterior and middle thirds. The lesion exhibited well-defined borders and a whitish-yellow coloration, with a more intense hue at the center. It measured approximately 1 cm in diameter, was mobile, firm on palpation, asymptomatic, and covered by smooth mucosa (Figure 1A). Extraoral examination revealed no palpable cervical lymph nodes and no associated facial or cutaneous abnormalities.

Aspiration of the lesion yielded no contents. Based on its clinical characteristics, a presumptive diagnosis of lipoma versus leiomyoma was made, and surgical excision was planned. Preoperative laboratory investigations, including complete blood count, bleeding time, prothrombin time, and activated partial thromboplastin time, were all within normal limits.

**Figure 2:**
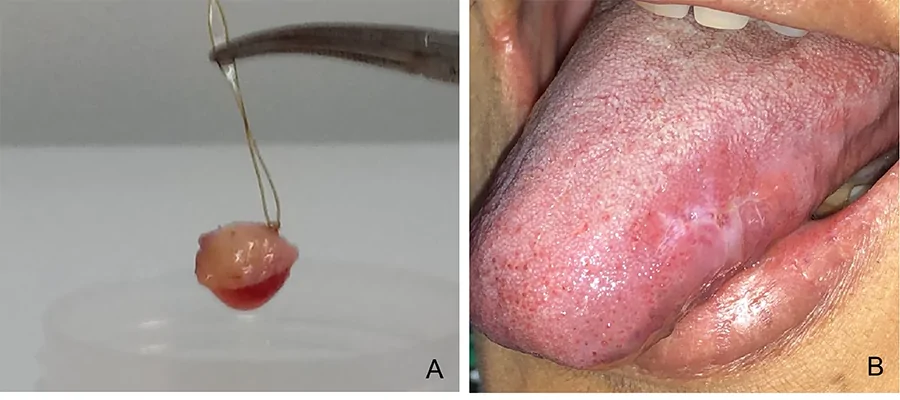
Encapsulated, yellowish excised specimen marked with a suture at the mesial end. (A) Gross specimen. (B) Follow-up at eight days post-suture removal.

Under local anesthesia, administered via a left mandibular block using 2% lidocaine with epinephrine (1:100,000), a longitudinal incision was made over the mucosa covering the tumor. The lesion appeared encapsulated, yellowish in color, and soft in consistency. It was easily enucleated without involvement of the lingual musculature. Meticulous hemostasis was achieved, and the surgical site was closed with simple interrupted sutures using 5-0 chromic catgut. The excised specimen, marked with a suture at its mesial end, was fixed in 10% neutral buffered formalin and submitted for histopathological examination (Figure 2A). Postoperative instructions emphasized the maintenance of proper oral hygiene. The patient was prescribed Enantyum® 25 mg (dexketoprofen), one tablet every eight hours for three days, and Oddent® gingival gel (containing chlorhexidine digluconate 0.20% and panthenol 1.0%), to be applied three times daily for five days. At the eight-day postoperative follow-up, the surgical site demonstrated satisfactory healing and favorable clinical progress (Figure 2B).

**Figure 3:**
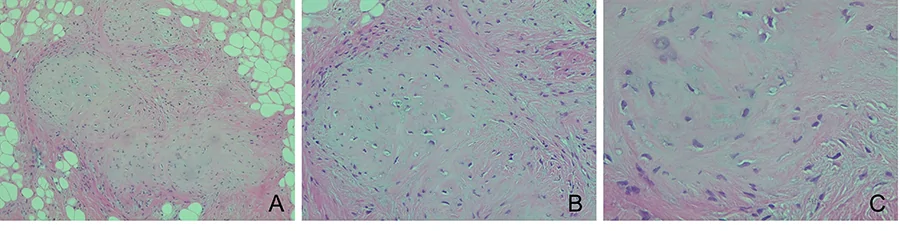
Histopathological findings. (A) Well-defined lesion composed of mature hyaline cartilage, partially surrounded by mature adipose tissue (H&E, 10×). (B) Central area showing uniform chondrocytes in lacunae within a chondroid matrix, without atypia or mitotic activity (H&E, 20×). (C) Higher magnification of the same area (H&E, 40×). H&E: hematoxylin and eosin.

Histopathological examination revealed a well-circumscribed proliferation of mature hyaline cartilage composed of uniform chondrocytes arranged in lacunae within a chondroid matrix. Mature adipose tissue was present adjacent to the lesion, with no evidence of tumor infiltration. No cellular atypia, mitotic activity, or features suggestive of malignancy were identified. The surgical margins were free of the lesion. These findings were consistent with a diagnosis of cartilaginous choristoma (Figure 3).

**Figure 4:**
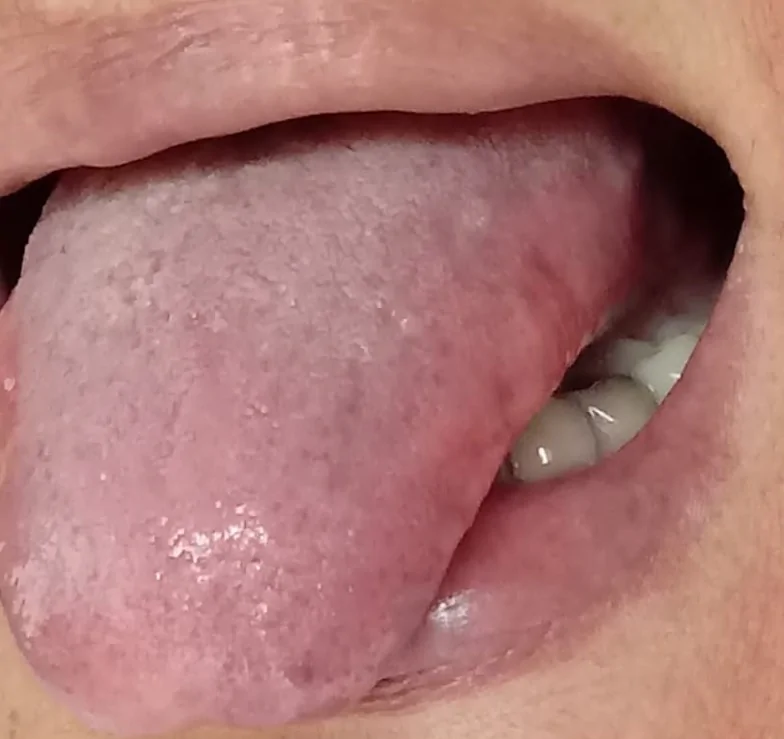
Five-year follow-up, frontal view.

Clinical follow-up was conducted over a five-year period, based on intraoral examinations that included visual inspection, palpation, and assessment of tongue mobility, strength, and sensation. No recurrence was observed, the lingual musculature remained unaffected, and the patient retained full motor and sensory function of the tongue (Figure 4). 

Written informed consent was obtained from the patient for publication of this case.

## DISCUSSION

The present case demonstrates features consistent with those reported in the literature, which indicate that the age of onset of cartilaginous choristomas varies widely, ranging from childhood to advanced age (5-73 years). The sex distribution remains unclear; however, a slight female predilection has been suggested ^7^, aligning with the characteristics of our patient.

The most common intraoral sites for cartilaginous choristomas are the dorsum and lateral borders of the tongue. This predilection may be associated with the embryologic development of the tongue, as heterotopic cartilaginous remnants can persist during the fusion of the first branchial arches and later proliferate. Alternatively, cartilaginous metaplasia may occur secondary to chronic irritation or trauma ^1^^,^^12^. In the present case, no history of trauma or other predisposing factors was identified.

Typically, these lesions present as well-circumscribed, firm, and mobile submucosal nodules covered by normal mucosa, without ulceration or pain ^2^^,^^6^. Their diameter usually ranges from 0.5 cm to 4.5 cm ^13^, and they may remain clinically stable for years, potentially delaying diagnosis ^7^. The present case exhibited many of these characteristics, emphasizing the importance of maintaining a high index of clinical suspicion-even for small lesions-as early recognition enables timely intervention with minimal morbidity.

It is essential to differentiate cartilaginous choristoma from other, more common tongue lesions. Fibromas are typically softer and often associated with chronic trauma or biting ^14^. Lipomas exhibit a more elastic consistency and a characteristic yellowish coloration ^15^. Leiomyomas, originating from smooth muscle, tend to present as firmer lesions that may occasionally be painful ^10^. Neurofibromas are generally diffuse and non-encapsulated ^16^, while granular cell tumors usually display a granular or elevated surface texture ^17^. In the present case, the lesion was clinically suggestive of either a lipoma or a leiomyoma, emphasizing that a definitive diagnosis relied on histopathological evaluation. Microscopic examination revealed well-differentiated mature cartilage without evidence of atypia or mitotic activity, surrounded by fibrous connective tissue and, in some areas, adjacent adipose tissue without signs of infiltration-findings consistent with a non-neoplastic, ectopic tissue origin ^7^. These features confirmed the diagnosis of a lingual cartilaginous choristoma.

The treatment of choice for lingual cartilaginous choristomas is complete surgical excision, ensuring removal of the lesion while preserving the lingual musculature and maintaining motor and sensory functions ^1^. These lesions are benign, and recurrence is exceedingly rare; therefore, the postoperative prognosis is excellent ^6^. Complete resection with preservation of the surrounding tissues typically results in rapid recovery, minimal morbidity, and full preservation of tongue function ^13^. In the present case, the patient demonstrated satisfactory healing and no recurrence during a five-year follow-up period, consistent with previously reported findings in the literature.

This case provides practical insights into the recognition, diagnosis, and management of lingual cartilaginous choristoma. By describing its clinical presentation, surgical removal, and five-year follow-up, it highlights important considerations for clinical practice, including functional assessment and postoperative monitoring. However, its main limitation lies in the scarcity of reported instances involving oral soft tissues, which hampers the establishment of clear clinical patterns and the optimization of treatment strategies.

## CONCLUSIONS

Accurate diagnosis of lingual cartilaginous choristoma is essential for ensuring appropriate and timely management. Therefore, it should be considered in the differential diagnosis of tumorous lesions affecting the soft tissues of the oral cavity, despite its low incidence. It is crucial that healthcare professionals (particularly dentists) be well acquainted with the clinical characteristics of this rare lesion.
